# Placental Sonomorphologic Appearance and Fetomaternal Outcome in Fontan Circulation

**DOI:** 10.3390/jcm13175193

**Published:** 2024-09-01

**Authors:** Elena Jost, Ulrich Gembruch, Martin Schneider, Andrea Gieselmann, Karl La Rosée, Diana Momcilovic, Christian Vokuhl, Philipp Kosian, Tiyasha H. Ayub, Waltraut M. Merz

**Affiliations:** 1 Department of Obstetrics and Prenatal Medicine, University Hospital Bonn, Venusberg-Campus 1, 53127 Bonn, Germany; ulrich.gembruch@ukbonn.de (U.G.); philipp.kosian@ukbonn.de (P.K.); tiyasha_hosne.ayub@ukbonn.de (T.H.A.); waltraut.merz@ukbonn.de (W.M.M.); 2 Department of Cardiology, German Paediatric Heart Centre, University of Bonn, Venusberg-Campus 1, 53127 Bonn, Germany; martin.schneider@ukbonn.de (M.S.); andrea.gieselmann@ukbonn.de (A.G.); 3 Clinic for Cardiology, ‘Kardio Bonn’, Baumschulallee 1, 53115 Bonn, Germany; larosee@kardio-bonn.de; 4 Department of Cardiology and Pulmonology, University Hospital Bonn, Venusberg-Campus 1, 53127 Bonn, Germany; diana.momcilovic@ukbonn.de; 5 Section of Pediatric Pathology, Department of Pathology, University Hospital Bonn, Venusberg-Campus 1, 53127 Bonn, Germany; christian.vokuhl@ukbonn.de

**Keywords:** Fontan circulation, placenta, high-risk pregnancies, placental sonomorphology, placental histopathology, uteroplacental perfusion, fetal growth restriction, ACHD, congenital heart disease

## Abstract

**Objectives:** Pregnancies in women with Fontan circulation are on the rise, and they are known to imply high maternal and fetal complication rates. The altered hemodynamic profile of univentricular circulation affects placental development and function. This study describes placental sonomorphologic appearance and Doppler examinations and correlates these to histopathologic findings and pregnancy outcomes in women with Fontan circulation. **Methods:** A single-center retrospective analysis of pregnancies in women with Fontan circulation was conducted between 2018 and 2023. Maternal characteristics and obstetric and neonatal outcomes were recorded. Serial ultrasound examinations including placental sonomorphologic appearance and Doppler studies were assessed. Macroscopic and histopathologic findings of the placentas were reviewed. **Results:** Six live births from six women with Fontan physiology were available for analysis. Prematurity occurred in 83% (5/6 cases) and fetal growth restriction and bleeding events in 66% (4/6 cases) each. All but one placenta showed similar sonomorphologic abnormalities starting during the late second trimester, such as thickened globular shape, inhomogeneous echotexture, and hypoechoic lakes, resulting in a jelly-like appearance. Uteroplacental blood flow indices were within normal range in all women. The corresponding histopathologic findings were non-specific and consisted of intervillous and subchorionic fibrin deposition, villous atrophy, hypoplasia, or fibrosis. **Conclusions:** Obstetric and perinatal complication rates in pregnancies of women with Fontan circulation are high. Thus, predictors are urgently needed. Our results suggest that serial ultrasound examinations with increased awareness of the placental appearance and its development, linked to the Doppler sonographic results of the uteroplacental and fetomaternal circulation, may be suitable for the early identification of cases prone to complications.

## 1. Introduction

Fontan circulation is the result of a multi-step surgical treatment for several types of congenital heart diseases (CHD). Introduced in 1986, refinements in the surgical treatment allow affected individuals a higher life expectancy and quality of life [1]. As a result, affected female adults with congenital heart disease (ACHD) may aim at pursuing pregnancy. In a Norwegian cohort study, the prevalence of CHD in women of reproductive age was 34.5 per 10,000, and the overall rate of deliveries in women with CHD was 29.5 per 100,000 [2]. According to data from the US, the rate of deliveries in women with Fontan circulation increased from 2.4 per million in 2000 to 30.3 per million in 2018 [3].

The hemodynamic changes of pregnancy pose a challenge to all with ACHD. Accordingly, cardiac event rates are increased [4,5,6,7]. In addition, obstetric and fetal/perinatal complications occur more frequently [5,8,9,10,11,12,13,14,15,16].

As hemodynamic changes are most pronounced in Fontan circulation, pregnancies are generally regarded as very high risk or even contraindicated [5,17,18]. However, an increasing number of publications report successful outcomes and low cardiac complication rates in selected cases [4,5,11].

The hemodynamic features of univentricular circulation include elevated venous pressure, low oxygen concentration, altered rheologic features, and changes in the coagulation profile [1,15,19,20]. These may impact placental development and function. In addition, medication may affect uteroplacental perfusion and hemostasis [21].

We hypothesized that the peculiarities of univentricular circulation would impact the uteroplacental perfusion, resulting in distinct ultrasound and histomorphologic features and affecting obstetric and fetal/perinatal outcome. For this, we reviewed all cases with Fontan circulation who were cared for at our institution.

## 2. Materials and Methods

Cases of women with Fontan circulation who presented at our institution for either preconception/contraceptive counseling or maternity care from 2018 to 2023 were extracted from our institutional database. Risk-adapted maternal and fetal surveillance was performed by our pregnancy heart team. For all pregnant patients, medical history, outcome, and ultrasound findings were retrieved from the hospital information system ORBIS (Dedalus Healthcare, Bonn, Germany) and the ultrasound software ViewPoint™ (Version 5 and 6, GE HealthCare, Solingen, Germany).

Prenatal examinations included a detailed obstetric ultrasound including fetal echocardiography and Doppler ultrasound examinations of the fetal, fetomaternal, and uteroplacental circulation. The modified WHO score (mWHO) was applied for risk classification [17], and maternal cardiac surveillance was performed accordingly.

The special focus of this study was the placental sonomorphologic appearance and development over time. The localization of the placenta, its thickness, echogenicity, presence of placental lakes or calcifications, and umbilical cord insertion were routinely recorded by qualified senior obstetric ultrasound experts during antenatal visits and stored as 2D images and videos. Serial fetal biometry and Doppler sonographic examinations of the uteroplacental system were performed including the pulsatility indices (PI) of the uterine arteries and umbilical artery. Abnormal results were defined as PI > 95th percentile.

After delivery, photo documentation of the fresh placenta and after histopathologic fixation was performed. Histopathologic findings were described according to the Amsterdam classification system [22].

Maternal outcome parameters included obstetric and cardiac complications, delivery details, duration of inpatient stay after delivery, intensive care unit admission, and breastfeeding.

Standard parameters of perinatal outcomes including neonatal intensive care unit (NICU) admission were assessed. Prematurity was defined as birth <37 + 0 weeks of gestation (WoG). Small for gestational age (SGA) was defined as estimated fetal weight or birthweight < 10th percentile. Fetal growth restriction (FGR) was defined as estimated fetal weight or birthweight < 10th percentile or fetal growth arrest in combination with abnormal Doppler results of the uterine or umbilical arteries and/or oligohydramnios [23].

## 3. Results

**Figure 1:**
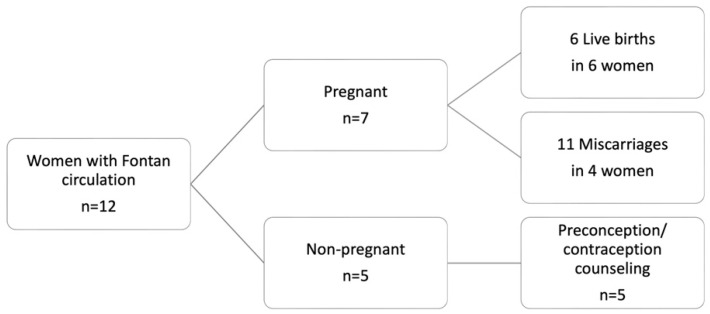
Overview of women with Fontan circulation and resulting pregnancies, who presented at the study center between 2018 to 2023.

From 2018 to 2023, 12 women with Fontan circulation presented at our center for co-care. Five of these women received preconception/contraceptive counseling and did not conceive during the observation period. The remaining seven women had 17 pregnancies, which resulted in six livebirths in six different women and 11 miscarriages (Figure 1). Eight miscarriages occurred during the first trimester, with three between 12 and 24 WoG. In the following study, only pregnancies resulting in livebirths are presented.

### 3.1. Maternal Characteristics, Obstetric and Neonatal Outcomes

Findings before and during pregnancy and before discharge after inpatient stay for delivery are presented in Table 1. The mean maternal age at delivery was 31.6 years (range 28–39). The mean pre-pregnancy BMI was 24.2 kg/m^2^ (range 21.2–25.9). All women were primiparous; three patients had a history of 1–4 miscarriages. Only the most recent cases (4, 5, and 6) presented for preconception counseling, while case 1 initially presented during the late second trimester at our department.

All the women had a left systemic ventricle following a modified Fontan procedure (i.e., cavopulmonary anastomosis) [24]. All but one case were considered mWHO III, whereas case 5 was classified as mWHO IV due to a history of transient ischemic attacks and Fontan-associated liver disease (FALD) [17]. Four women were on prophylactic dosages of low molecular weight heparin (LMWH), one on an intermediate dosage in combination with acetylsalicylic acid (ASA) until 30 WoG, and one woman received only ASA during pregnancy. Prior to pregnancy, two women were on phenprocoumon (changed to prophylactic LMWH in week 5), two on direct oral anticoagulants (changed to prophylactic LMWH in week 6), and two on ASA. All women except case 6 were on a ß-blocker, which was continued during pregnancy in case 4 and 5.

Systemic ventricular function based on serial transthoracic echocardiograms remained stable in all cases throughout the pregnancy. Furthermore, SpO_2_ baseline levels pre-pregnancy were >95%, and remained stable in half of the patients (3/6) but dropped to 88–92% in the remaining women during pregnancy and the early postpartum period. Major cardiovascular complications did not occur during the study period. One woman suffered a transient ischemic attack 3 months postpartum (case 5).

Four patients (66%) delivered via cesarean section (CS), which were all due to obstetric indications. FGR during pregnancy was suspected in 4/6 cases (66%). All but one woman delivered prematurely, accounting for a rate of 83%. The mean gestational age (GA) at delivery was 34 + 2 WoG (range 27 + 5–38 + 1). Birthweight < 10th percentile was present in three of six newborns, with a mean birthweight of 1943 g (range 930–2690), corresponding to the 15th percentile (range 5th–36th). Four out of six women (66%) suffered bleeding complications, and two required transfusions. The mean duration of postpartum inpatient stay was 7.7 days (5–12 days).

The neonatal outcomes were good in all cases. The majority of neonates (4/6) were admitted to the NICU due to prematurity with a mean duration of 24 days (range 8–67 days).

### 3.2. Placental Findings

**Figure 2:**
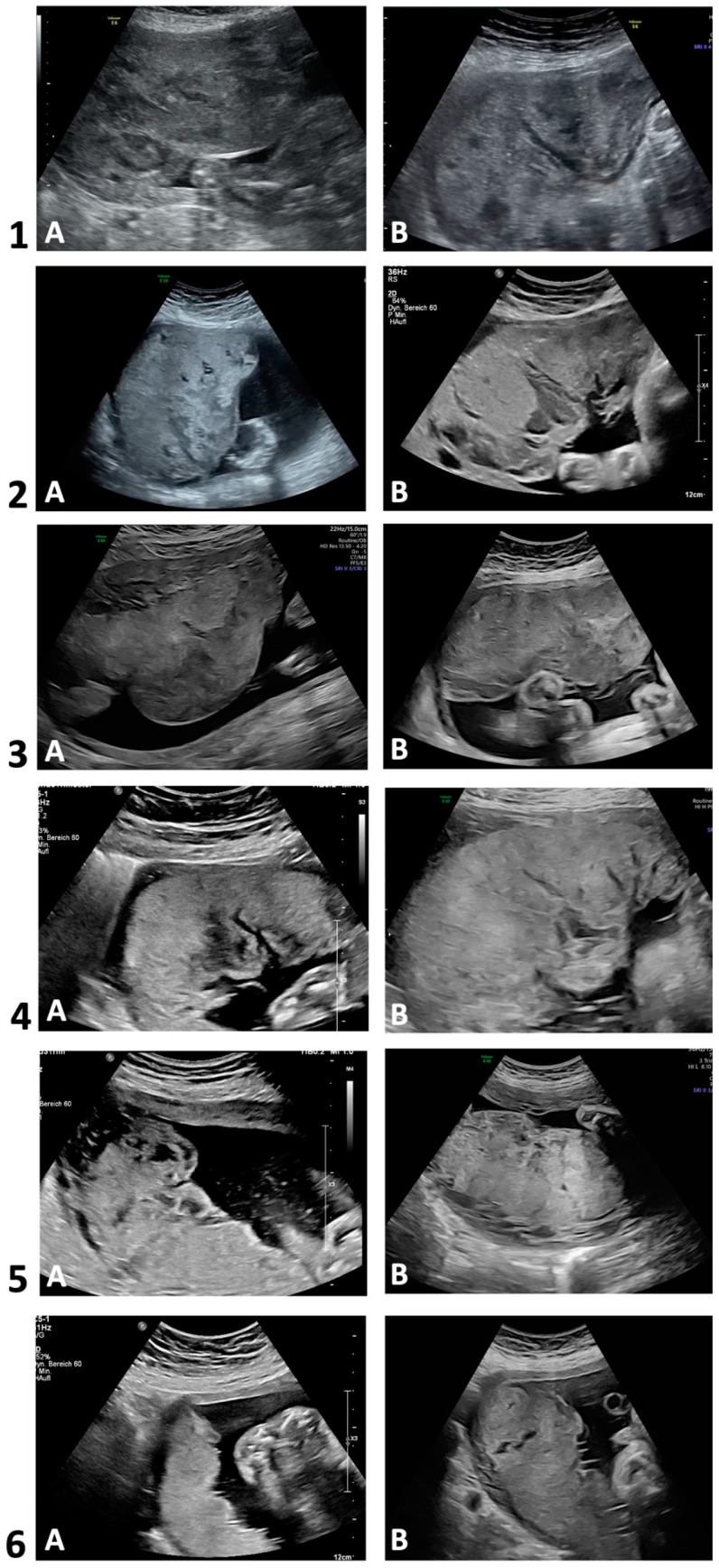
Transabdominal ultrasounds in 2D grayscale. Development of placental sonomorphologic appearance of cases with Fontan circulation (n = 6). Case 1 (A: 25 + 5; B: 32 + 1), case 2 (A: 29 + 4; B: 36 + 3); case 3 (A: 20 + 5; B: 30 + 5); case 4 (A: 23 + 6; B: 25 + 0); case 5 (A: 22 + 1; B: 32 + 1); case 6 (A: 24 + 0; B: 34 + 6). Gestational age (week + day).

Figure 2 depicts two placental ultrasound images per case to demonstrate the sonomorphologic appearances. Table 2 summarizes the ultrasonographic morphology in each trimester. Sonographic alterations in placental appearance could be initially detected during first trimester (13 WoG) in one out of four examined pregnancies (case 3), in four cases during the late second trimester (22–26 WoG), and in case 6 during the third trimester (35 WoG). In cases 1–5, the placentas in the second and third trimester were markedly thickened and interspersed with large lakes, impressing as homogeneous anechoic or hypoechoic areas surrounded by placental tissue of normal echogenicity. The mostly very large lakes were localized both centrolobularly and subchorionically, as well as marginally, partly extending from the basal plate to below the chorion, and conveyed a jelly-like appearance of the entire placenta with slow or sonographically undetectable blood flow [25]. In the third trimester, the size of some of the lakes increased, and in two cases, the large subchorionic lakes were also partially thrombosed. Case 5 additionally showed hyperechogenic calcifications scattered throughout the placenta in both the second and third trimesters. In case 6, however, the placenta was neither thickened nor did it show abnormal echogenicity in the second trimester. In the third trimester, however, a few small centrolobularly located lakes with hyperechogenic rims were present.

In the Supplementary Materials, an ultrasound video of a third-trimester placenta (case 2, 36 + 3 WoG) illustrates the above-mentioned alterations (Supplementary Materials, Video S1). For a correlation of the sonographic findings with macroscopic appearance, a photo of the same placenta one day later is depicted in Figure S1 of the Supplementary Materials.

**Figure 3:**
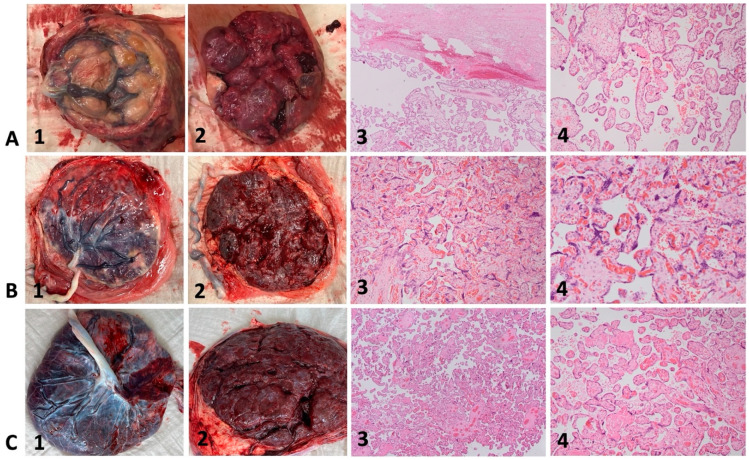
Macroscopic and microscopic placental appearance in cases with Fontan circulation (**A**,**B**) and control (**C**). Hematoxylin-eosin staining with magnification 40× (column 3) and 80× (column 4). 3A–C (1) fresh placentas, fetal surface; 3A–C (2) fresh placentas, maternal surface; 3A–C (3 + 4) representative histologic images of corresponding placental specimens (Hematoxylin-eosin staining, magnification 40× and 80×). 3A, 1–4: Case 4, 27 + 5: small, globular shape, circumvallate membranes, and extensive subchorionic fibrin deposition; subchorionic fibrin (3A, 3, upper part) and mainly terminal villi can be observed. 3B, 1–4: Case 6, 38 + 1: few scattered areas of calcification; syncytial knots. 3C, 1–4: Healthy control; normal macroscopic and microscopic appearance at term after uneventful pregnancy.

As an example, the macroscopic appearance of three fresh placentas and representative histopathologic images of corresponding placental specimens are illustrated in Figure 3. The placenta of case 4 (Figure 3A, 1–4) shows placental pathology such as small, globular shape, circumvallate membranes, and extensive subchorionic fibrin deposition. Microscopically, subchorionic fibrin (Figure 3A, 3, upper part) and mainly terminal villi can be observed. The placenta of case 6 (Figure 3B, 1–4) shows less placental pathology with only a few scattered areas of calcification. Microscopically, mainly tertiary villi with syncytial knots can be observed.

An overview of the histopathologic findings is displayed in Table 2. Placental weight was below the third percentile in 50% (3/6) of cases. Macroscopically, 2/6 placentas showed marginal insertion and small vessel caliber of the umbilical cord. Subchorionic fibrin deposition was found in 4 out of 6, and subchorionic hematoma was present in 3/6 placentas. None of the placentas showed signs of infarction. Two out of six presented with a placenta circumvallate. Microscopically, five out of six placentas showed intervillous fibrin deposits, 2/6 villous fibrosis, and 2/6 villous atrophy. Villous hypoplasia was found in 1/6 placentas, syncytial knots in 2/6, and parenchymal hematoma in 2/6 placentas.

### 3.3. Fetal Growth Assessment and Doppler Studies

**Figure 4:**
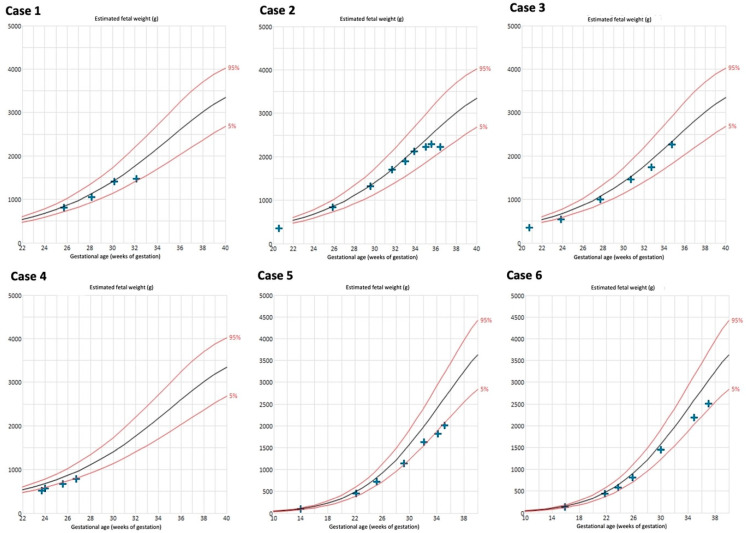
Estimated fetal weight measurements of cases with Fontan circulation (n = 6). Reference curves of 5th (lower red line), 50th (black line), and 95th percentile (upper red line) [27,28].

The biometry results are depicted in Figure 4. Late onset FGR developed in 4/6 cases (66%). Case 4 delivered at a very early GA; FGR during the further course of pregnancy may have occurred. Birthweight <10th percentile was present in 50% (3/6) of the neonates (Table 1).

**Figure 5:**
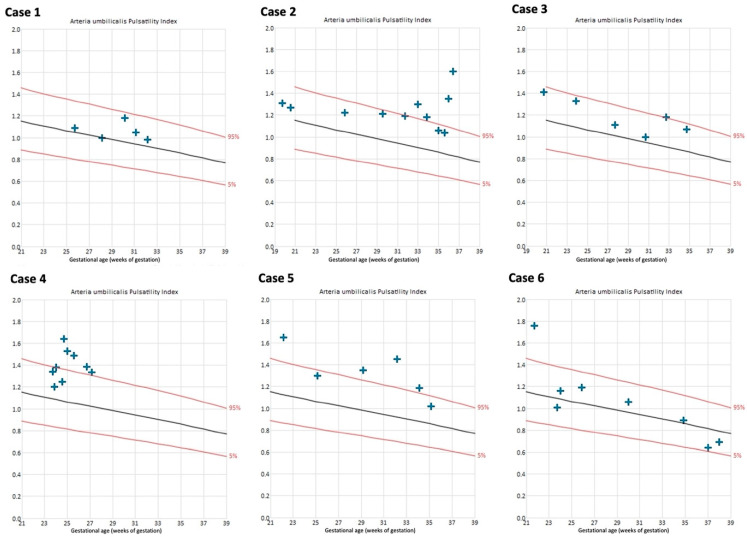
Umbilical artery pulsatility indices of cases with Fontan circulation (n = 6). Reference curves of 5th (lower red line), 50th (black line), and 95th percentile (upper red line) [29].

The uterine artery PIs were within the normal range in all cases throughout the course of pregnancy, except for the left uterine artery in case 4 (Figure S3 of the Supplementary Materials). Umbilical artery PIs remained within normal range in 4/6 cases (Figure 5).

## 4. Discussion

The results of our retrospective single-center series of pregnancies in Fontan circulation confirm previous reports with respect to the maternal, obstetric, and neonatal outcomes. Our detailed examinations of the placenta morphology and function offers an explanation for the high obstetric and fetal/perinatal complication rates and may serve as a blueprint for surveillance.

We assume the excellent maternal cardiac outcomes are the result of the stable preconception cardiac function and close monitoring during pregnancy. Similar results have been published [5,11], including a systematic literature review by Garcia Ropero et al. (2018) [4].

Obstetric and fetal/perinatal complications are increased in all ACHD women. These include miscarriage (19.4%), prematurity (OR 1.8), FGR (OR 2.3), preeclampsia (OR 2.6), and delivery by CS [5,8,9]. In pregnancies with Fontan circulation, complication rates are even higher. Early and late miscarriage rates of 26–67% (16%, respectively) have been reported [4,10,11]. Intrauterine fetal demise (IUFD) occurs in 3–5% of pregnancies [10,11]. Furthermore, bleeding events are particularly common in women with univentricular circulation. In a retrospective study with 108 pregnancies, antepartum placental hematoma, placental abruption, or postpartum hemorrhage occurred in 35.2% of cases, with 13% considered life-threatening [12]. In the case series by Cauldwell et al. (2016), Fontan physiology was associated with the highest blood loss of all CHD types [13]. With respect to perinatal complications, prematurity or FGR occur in 38–82% of pregnancies [10,11,14,15,16]. Our results are in accordance with these findings.

In analogy to other intraabdominal organs, the placenta is exposed to the peculiarities of Fontan physiology. These are summarized by Siegmund et al. (2022) and include the following: forward and backward failure; reduced capacity to adapt during exercise; elevated venous pressure with concomitant venous stasis provoking chronic placental abruptions with consecutive subchorionic fibrin deposition; low oxygen concentration; altered rheologic features; and changes in the coagulation profile [1,15,19,20]. Alterations in placental development and function can therefore be expected. We observed highly abnormal placental sonomorphology, which evolved during the course of pregnancy in the majority of our patients. The placentas were markedly thickened and had a jelly-like appearance [25]. Interspersed lakes were present with slow or absent blood flow; partial thrombosis could be detected during the further course of pregnancy. Our findings confirm the results of previous publications. Putman et al. (2023) described similar findings in three women [30], and Ordoñez et al. (2021) in a single case [31]. It may be envisaged that these alterations contribute to the high risk of bleeding during any time of pregnancy and delivery. Heparin and/or ASA medication further increase the chance of bleeding.

The manifestation of FGR may be another consequence of altered placental development. In our study, a prenatal diagnosis of FGR was established in 66% of cases; birthweight <10th percentile was present in 50% of the neonates. FGR in Fontan circulation has already been described. An estimated fetal weight <10th percentile was present in 23.1% of 104 cases published by Girnius et al. (2021); birth weight percentiles are not mentioned [12]. Of 13 neonates (31%) of women with Fontan circulation, 4 reported by Philipps et al. (2019) were labelled as SGA, without further specification [32]. Older reports are summarized in a systematic review by Garcia Ropero et al. (2018). Here, 7 of 115 live births (20%) were SGA, without specification. Underreporting may have occurred since severe, early-onset FGR may result in the termination of pregnancy or IUFD, which happened in 21 cases [4].

In the majority of cases, FGR develops as a sequela of impaired uteroplacental blood flow. The diagnosis is based on abnormal Doppler indices of the uterine arteries during mid-gestation. However, Doppler indices of the uteroplacental perfusion were within normal range in all our patients. We observed abnormal Doppler indices of the umbilical artery as an indication of fetal malperfusion in the placental villi in 50% of cases. Therefore, the favored hypothesis of impaired extravillous invasion and transformation of the spiral arteries seems unlikely. Based on the highly abnormal placental findings, we hypothesize a primary intraplacental origin. Primary maternal uteroplacental perfusion disorders, for example, due to increased venous pressure and reduced cardiac ejection, present in single ventricle palliation may impair placental growth and development. In the development of both PE and FGR, pre-existing maternal cardiovascular dysfunction and cardiovascular maladaptation to the increased stress of pregnancy are discussed [33,34], but also with a disruption of venous outflow of the intervillous space by inadequate adaption of uterine venous circulation or pre-existing maternal venous hemodynamic dysfunction with venous congestion [35] as possible primary causes. In addition, the detected histopathological changes correspond to those of maternal vascular malperfusion (massive inter- and perivillous fibrin deposits, abnormalities of villous development as distal villous hypoplasia, and accelerated villous maturation with small or short hypermature villi for the gestational period accompanied by an increase in syncytial nodules) [22]. On the other hand, there were no histopathological signs of fetal vascular malperfusion or villitis of unknown etiology [22]. The extensive subchorionic and/or intervillous fibrin deposits found in most of placental specimens as well as villous atrophy, hypoplasia, or fibrosis may impair transplacental substrate exchange. We were able to recapitulate histopathologic findings in placentas of women with Fontan physiology published so far [19,32,36,37,38]. In a retrospective single-center study, Wu et al., (2022) analyzed placentas of women with various CHD, including six cases of Fontan circulation, showing unusual prominence of subchorionic fibrin deposition. They attributed impaired placental function and fetal perfusion to this finding [36]. Excessive subchorionic fibrin deposition was also described in the analysis of seven pregnancies in women with Fontan circulation by Phillips et al., in 2019 [32]. The case series of eight pregnancies by Yokouchi-Konishi et al. (2021) additionally outlined the aspect of placental hypoxic lesions as a potential reason for poor pregnancy outcomes in affected women [38]. Overall, placental abnormalities found in our study and published case series are very variable. Specific pathognomonic changes in placentas of women with Fontan circulation could not be defined. However, the findings all correspond to morphological changes that are caused by systemic hypoxemia and low placental perfusion.

The limitations of our study are the small number of cases and the retrospective design. Additionally, the comparison of placental sonomorphologic appearance, histopathologic characteristics and their correlated fetomaternal outcome, is not performed in cases of miscarriage. However, serial fetomaternal assessments were performed, and a synopsis of maternal, obstetric, and perinatal data including ultrasound and Doppler studies as well as histopathological placental findings were available for analysis.

## 5. Conclusions

Adverse pregnancy outcomes are common in women with Fontan physiology. Therefore, predictors are urgently needed. Our results suggest that serial ultrasound examination of the placental appearance combined with Doppler studies of the uteroplacental and fetomaternal blood flow may be a suitable instrument for case identification. Further studies elucidating the peculiarities of Fontan physiology during pregnancy are required.

## Figures and Tables

**Table 1:** Maternal characteristics and obstetric, cardiac, and neonatal outcomes of livebirths in women with Fontan circulation (n = 6).

Case No.	1	2	3	4	5	6
Congenital heart disease	Double inlet LV with L-transposition of the great vessels	TA type Ib, pulmonary stenosis	PA/IVS	TA type Ib	Dextrocardia, single inlet LV with TGA position, single AV valve, asplenia, right atrial and abdominal isomerism	TA type Ib, PA, restrictive VSD
Type of repair	Modified Fontan	Modified Fontan	Modified Fontan	Modified Fontan	Modified Fontan	Modified Fontan
Comorbidities	-	-	Esophageal varices gradeI, s/p thrombosis of both femoral veins, arrhythmia (life vest)	s/p occlusion and recanalization of IVC, s/p pacemaker implantation	FALD, s/p TIA	FALD, trigonocephaly
mWHO classification	III	III	III	III	IV	III
Additional investigations during pregnancy	-	-	EGD in week 21	-	-	-
SpO_2_, % (bP/lowest iP/bD)	UNK */93/93	96/88/90	95/90/88	96/96/95	97/93/92	97/96/96
NT-pro-BNP, pg/mL (bP/highest iP/bD)	UNK */150/52	67/339 (week 36)/66	862/924 (week 31)/1676	87/80 (week 21)/21	166/155/240	69/71/68
Medication bP	Metoprolol, ASA	Captopril, ASA	Rivaroxaban (until week 6), bisoprolol	Phenprocoumon (until week 5), metoprolol	Apixaban (until week 6), metoprolol	Phenprocoumon (until week 5)
Medication iP	ASA	Intermediate LMWH, ASA (until week 30)	Prophylactic LMWH	Prophylactic LMWH, metoprolol	Prophylactic LMWH, metoprolol	Prophylactic LMWH
Preconception counseling	-	-	-	Yes	Yes	Yes
Gravida/Para	I/0	I/0	I/0	V/0	II/0	V/0
Obstetric findings	-	dGDM, breech	preeclampsia	Vaginal bleeding, dGDM	-	-
Indication for delivery	FGR	FGR	Preeclampsia	PPROM, preterm labor	FGR	PPROM, FGR
Mode of delivery	Elective CS	Elective CS	Elective CS	SVD	Elective CS	IVD
Type of anesthesia	Spinal	Spinal	Spinal	-	Spinal	Epidural
GA at delivery, week + day	32 + 6	36 + 4	34 + 6	27 + 5	36 + 0	38 + 1
Complications during delivery	PPH, suspected AIP	Placenta adhaerens	-	-	-	Placenta adhaerens
Blood loss (mL) and transfusion	1200/2 PRBC	800	400	300	300	500/2 PRBC
ICU stay pp	1 night	-	1 night	-	-	1 night
Inpatient stay for delivery, days (total/pp)	16/12	11/8	10/8	34/6	7/7	6/5
Birthweight, gram (percentile)	1685 (25%)	2190 (6%)	1970 (12%)	930 (36%)	2190 g (8%)	2690 (5%)
Apgar score, 1/5/10 min	9/10/10	10/10/10	8/9/9	7/9/9	9/10/10	7/8/10
Umbilical-artery pH/BD (mmol/L)	7.34/4.0	7.27/3.70	7.38/UNK	7.38/1.10	7.35/2.30	7.14/9.70
NICU stay, days	11	8	10	67	-	-
Breastfeeding	Yes	Yes	Yes	Yes	Yes	Yes
Maternal follow-up results	NAD	FALD (14 months pp)	NAD, NT-pro-BNP decreased to 900 pg/mL	NAD	TIA (3 months pp)	No FU data
Neonatal findings and follow-up results	NAD	NAD	NAD	PDA	No FU data	Trigonocephaly, no FU data

AIP: abnormally invasive placenta; ASA: acetyl salicylic acid; AV: atrioventricular; bD: before discharge after inpatient stay for delivery; bP: before pregnancy; CS: cesarean section; dGDM: dietary-controlled gestational diabetes mellitus; EGD: esophagogastroduodenoscopy; FALD: Fontan-associated liver disease; FGR: fetal growth restriction; FU: follow-up; GA: gestational age; iP: in pregnancy; IVC: inferior vena cava; LMWH: low molecular weight heparin; LV: left ventricle; min: minutes; mWHO: modified World Health Organization; NAD: no abnormality detected; (N)ICU: (neonatal) intensive care unit; PA: pulmonary valve atresia; PA/IVS: pulmonary atresia with intact ventricular septum; PDA: patent ductus arteriosus; pp: postpartum; PPH: postpartum hemorrhage; PPROM: preterm premature rupture of membranes; PRBC: packed red blood cells; s/p: status post; SpO_2_: pulse oximeter oxygen saturation; SVD: spontaneous vaginal delivery; TA: tricuspid valve atresia; TIA: transient ischemic attack; TGA: Transposition of the great arteries; UNK: unknown; IVD: instrumental vaginal delivery; VSD: ventricular septum defect. * Data not available due to initial presentation in late second trimester.

**Table 2:** Placental ultrasound and morphology (in reference to Figure 2, Figure 3 and Figure S2) of cases with Fontan circulation (n = 6).

Case No.	1	2	3	4	5	6
Ultrasound morphology (see Figure 2)						
- First trimester	No data	Normal size and echogenicity	Normal size and echogenicity except for some marginal lakes	No data	Normal size and echogenicity	Normal size and echogenicity
- Second trimester	Thickened placental shape; huge lakes distributed throughout the whole placenta (centrolobular, subchorionic and marginal), partially with central maternal clots (jelly-like appearance)	Thickened placental shape; huge lakes distributed throughout the whole placenta (centrolobular, subchorionic and marginal), (jelly-like appearance)	Thickened placental shape; huge lakes (subchorionic and marginal), (jelly-like appearance)	Thickened placental shape; huge lakes (subchorionic and marginal), (jelly-like appearance)	Thickened placental shape; some lakes (centrolobular, subchorionic and marginal), scattered intraplacental calcifications	Normal size and echogenicity
- Third trimester	Thickened placental shape; huge lakes distributed throughout the whole placenta (centrolobular, subchorionic), (jelly-like appearance)	Thickened placental shape; huge lakes distributed throughout the whole placenta (centrolobular, subchorionic and marginal), (jelly-like appearance)	Thickened placental shape; huge lakes (centrolobular, subchorionic and marginal), (jelly-like appearance)	Thickened placental shape; huge lakes (subchorionic and marginal), (jelly-like appearance)	Thickened placental shape; extensive lakes (centrolobular, subchorionic and marginal), scattered intraplacental calcifications (jelly-like appearance)	Normal echogenicity; only a few small centrolobular lakes with peripheral echogenicity
Macroscopic findings (see Figure 3 and Figure S2)						
- Placental weight, g (percentile range) [26]	383 (10–25th)	400 (<3rd)	360 (<3rd)	227 (3–10th)	383 (10–25th)	295 (<3rd)
- Umbilical cord abnormalities	-	Marginal insertion, small vessel caliber, congested blood	-	Marginal insertion, small vessel caliber, congested blood	-	-
- Placental shape	Fragmented parenchyma	Thick shape, rugged parenchyma	Large parenchymal lacunae	Small, thick shape, edematous areas	Regular	Regular
- Abnormalities of membranes	-	circumvallate	-	circumvallate	-	-
- Subchorionic fibrin deposition	-	-	yes	yes, extensive (25–33% of organ)	yes, extensive	yes
- Subchorionic hematoma	yes	yes	yes	-	-	-
- Signs of infarction	-	-	-	-	focal	-
Microscopic findings (see Figure 3)						
- Intervillous fibrin deposition	yes	yes	yes (5–10% of parenchyma)	yes	-	yes (<5% of parenchyma)
- Villous fibrosis	yes	yes	-	-	-	-
- Villous atrophy	-	yes	yes	-	-	-
- Villous hypoplasia	-	yes	-	-	-	-
- Syncytial knots	yes	-	-	-	-	-
- Parenchymal hematoma	yes	yes	-	-	-	-

## Data Availability

The original contributions presented in the study are included in the article/Supplementary Materials. Further inquiries can be directed to the corresponding author.

## Supplementary Materials

The following supporting information can be downloaded at: https://www.mdpi.com/article/10.3390/jcm13175193/s1, Figures S1–S3; Video S1. References [39,40] are mentioned in this part.

## Abbreviations

ACHD: adult congenital heart disease; ASA: acetylsalicylic acid; CHD: congenital heart disease; CS: cesarean section; FALD: Fontan-associated liver disease; FGR: fetal growth restriction; GA: gestational age; LMWH: low molecular weight heparin; NICU: neonatal intensive care unit; OR: odds ratio; PI: pulsatility index; SGA: small for gestational age; SpO_2_: pulse oximeter oxygen saturation levels; WoG: weeks of gestation.
