# Supplementary material for: Placental Sonomorphologic Appearance and Fetomaternal Outcome in Fontan Circulation
Source: J Clin Med. 2024 Sep 1;13(17):5193. doi: 10.3390/jcm13175193 (PMC11396425; doi:10.3390/jcm13175193)
Supplement: Supplementary file 1 [file jcm-13-05193-s001.zip › Supplementary material_22.05.2024.pdf]

Supplementary Materials

Figure S1

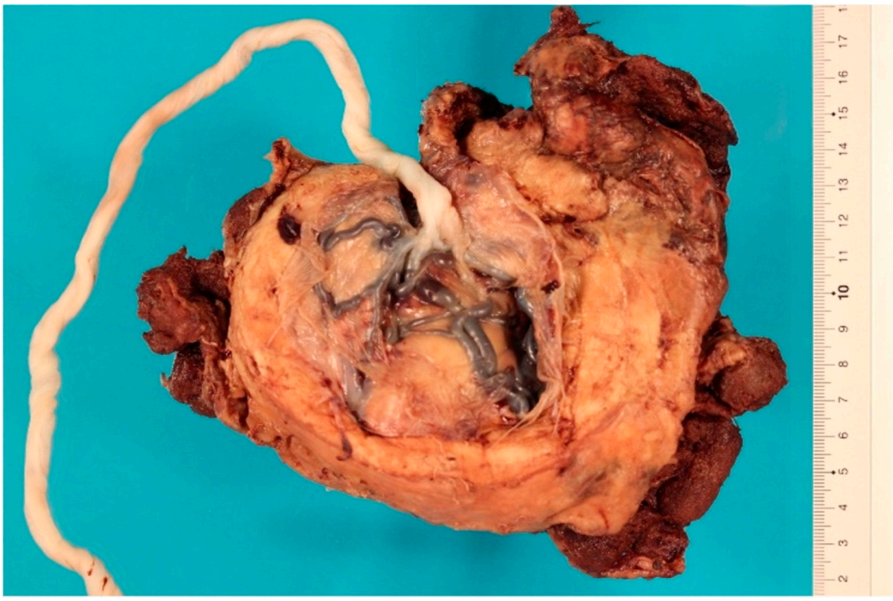

Figure S 1: Placenta case 2 (36w4d) after histopathologic fixation, which showed overall signs of pathology, namely thickened and globular shape, and extensive subchorionic fibrin deposition. In supplementary material video 1, sonographic appearance of this placenta was captured one day prior to delivery.

Figure S2

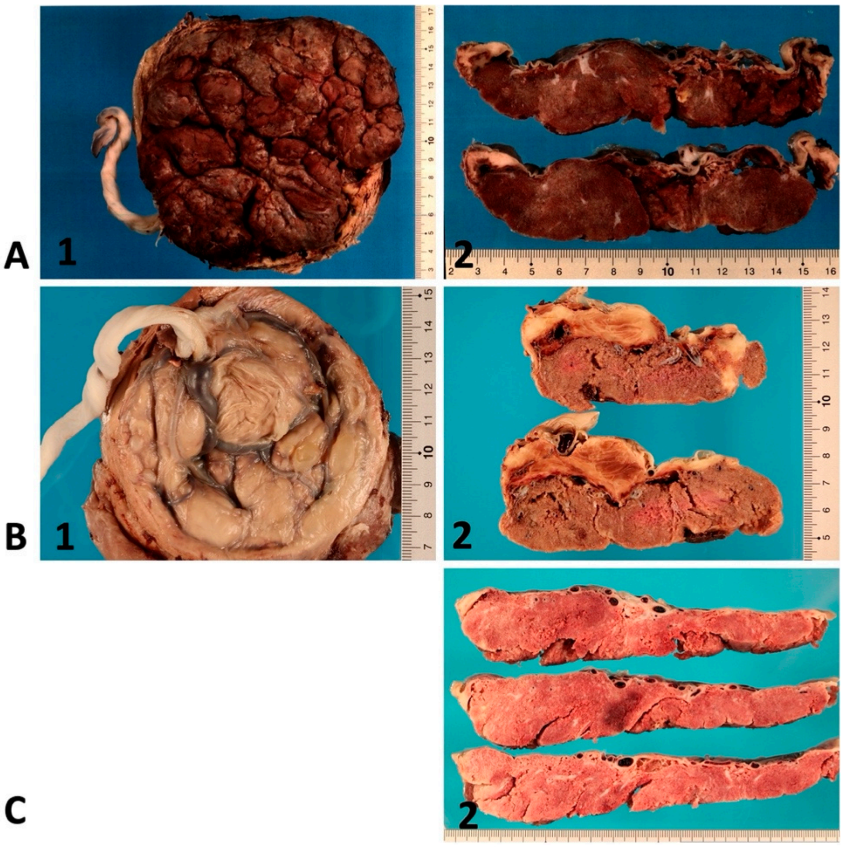

Figure S 2: Macroscopic placental appearance after histopathologic fixation. A 1+2: Case 3 (34+6): scattered calcifications and subchorionic hematoma. B 1+2: case 4 (27+5): placenta circumvallate with overall thick shape and extensive subchorionic fibrin deposition. C 2: healthy control (38+6). Gestational age (week+day).

Figure S3

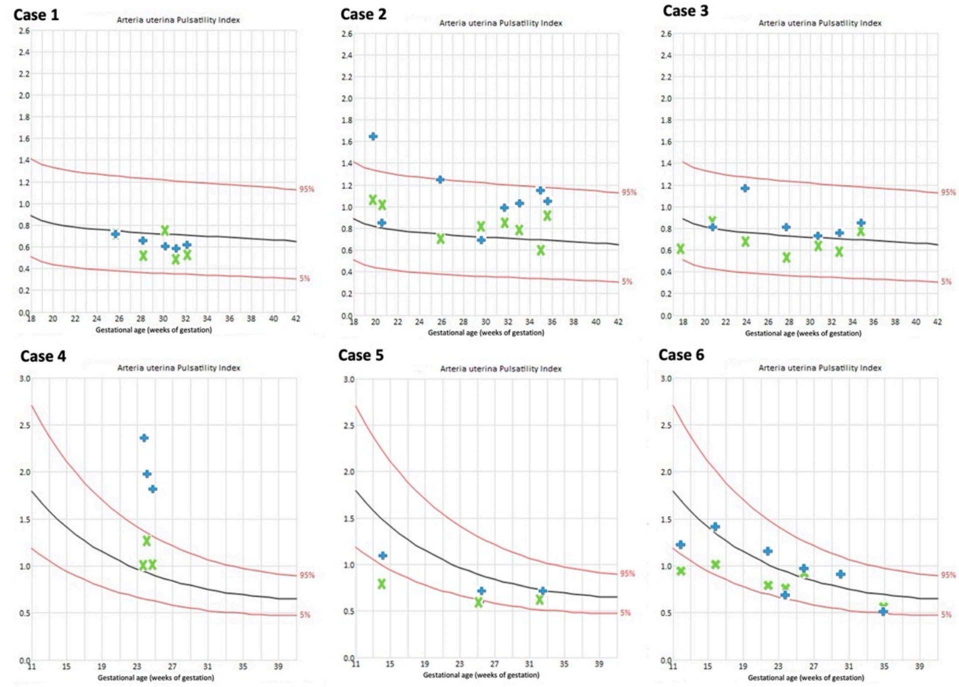

Figure S 3: Overview of the right (green cross) and left (blue plus sign) uterine artery Doppler flow measurements of each patient, depicted as pulsatility index (PI) over time in pregnancy (gestational age in weeks of gestation). Reference curves of 5th, 50th, and 95th percentile[1,2].

References

1. Bahlmann F, Fittschen M, Reinhard I, Wellek S, Steiner E. Reference Values for Blood Flow Velocity in the Uterine Artery in Normal Pregnancies from 18 Weeks to 42 Weeks of Gestation Calculated by Automatic Doppler Waveform Analysis. *Ultraschall in Med*. 2012;33(03):258-264. doi:10.1055/s-0031-1281647

2. Gómez O, Figueras F, Fernández S, et al. Reference ranges for uterine artery mean pulsatility index at 11–41 weeks of gestation. *Ultrasound in Obstet & Gyne*. 2008;32(2):128-132. doi:10.1002/uog.5315
